# High-Power Prismatic Devices for Oblique Peripheral Prisms

**DOI:** 10.1097/OPX.0000000000000820

**Published:** 2016-04-25

**Authors:** Eli Peli, Alex R. Bowers, Karen Keeney, Jae-Hyun Jung

**Affiliations:** *MS, OD, FAAO; ^†^PhD, FAAO; ^‡^MS; ^§^PhD; Schepens Eye Research Institute, Massachusetts Eye and Ear, Department of Ophthalmology, Harvard Medical School, Boston, Massachusetts (EP, ARB, J-HJ); and Chadwick Optical, Inc., Souderton, Pennsylvania (KK).

**Keywords:** low vision, vision rehabilitation, prism devices, field expansion, Fresnel prism

## Abstract

**Purpose:**

Horizontal peripheral prisms for hemianopia provide field expansion above and below the horizontal meridian; however, there is a vertical gap leaving the central area (important for driving) without expansion. In the oblique design, tilting the bases of both prism segments toward the horizontal meridian moves the field expansion area vertically and centrally (closing the central gap) while the prisms remain in the peripheral location. However, tilting the prisms results also in a reduction of the lateral field expansion. Higher prism powers are needed to counter this effect.

**Methods:**

We developed, implemented, and tested a series of designs aimed at increasing the prism power to reduce the central gap while maintaining wide lateral expansion. The designs included inserting the peripheral prisms into carrier lenses that included yoked prism in the opposite direction, combination of two Fresnel segments attached at the base and angled to each other (bi-part prisms), and creating Fresnel prism–like segments from nonparallel periscopic mirror pairs (reflective prisms).

**Results:**

A modest increase in lateral power was achieved with yoked-prism carriers. Bi-part combination of 36Δ Fresnel segments provided high power with some reduction in image quality. Fresnel reflective prism segments have potential for high power with superior optical quality but may be limited in field extent or by interruptions of the expanded field. Extended apical scotomas, even with unilateral fitting, may limit the utility of very high power prisms. The high-power bi-part and reflective prisms enable a wider effective eye scanning range (more than 15 degrees) into the blind hemifield.

**Conclusions:**

Conventional prisms of powers higher than the available 57Δ are limited by the binocular impact of a wider apical scotoma and a reduced effective eye scanning range to the blind side. The various designs that we developed may overcome these limitations and find use in various other field expansion applications.

In 2000, Peli^1^ proposed the use of peripheral prisms to expand the visual field of patients with homonymous hemianopia. The prism segments are placed peripherally on the spectacle carrier lens above and below the line of sight. They are usually applied unilaterally on the hemianopic (“blind”) side (Fig. 1A) and always with the base in the direction of the visual field defect. This method expands the binocular visual field as measured by perimetry (Fig. 1C) rather than merely shifting it, which is the case for binocular sector prisms.^3^

**FIGURE 1:**
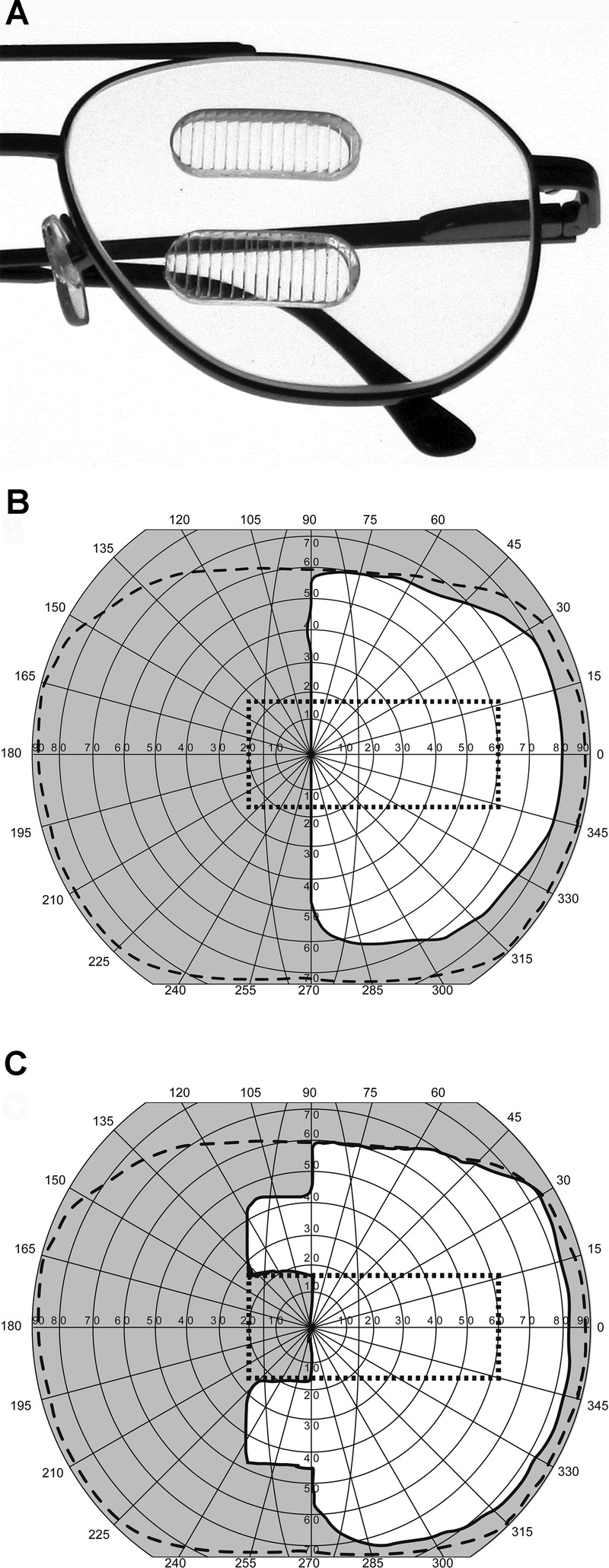
(A) Permanent peripheral prism glasses in the horizontal design constructed with embedded rigid PMMA Fresnel prism inserts of 36Δ above and below the pupil base-left on the left lens only for a patient with left hemianopia. (B) Binocular visual field of a patient with left hemianopia. (C) Binocular visual field of the same patient wearing horizontal peripheral prisms resulting in a field expansion of about 20 degrees. The outer dashed line represents the normal binocular visual field and the dotted rectangle represents the field of view through the windshield of a typical car.^2^

The central part of the spectacle carrier lens is prism-free, allowing single central binocular vision with the habitual distance prescription, if needed. It is the peripheral binocular visual confusion, two different objects seen in the same apparent direction,^1^,^3^ that provides the peripheral visual field expansion. Objects that would otherwise fall in the blind hemifield of the hemianopia-side eye are shifted to the residual seeing hemifield and become visible, superimposed on other objects seen by the corresponding retinal area in the seeing hemifield of the fellow eye. Although peripheral diplopia (seeing the same object in two different directions) can occur, it is minimal with this design. Because of the large apical scotoma associated with the high power of the peripheral prisms, much of the peripheral diplopia is avoided.^3^

The peripheral placement of the prisms enabled the use of higher-power prisms than in the earlier sector prism designs (usually <20Δ^4^), thereby providing greater field expansion. Initially, 40Δ, the highest power available in the temporary Fresnel press-on prisms (3M Inc., St. Paul, MN) was used,^1^ resulting in a lateral field expansion of about 22 degrees. Later, in collaboration with Chadwick Optical Inc. (Souderton, PA), permanent peripheral prism glasses using 40Δ rigid polymethyl methacrylate (PMMA) Fresnel prism segments (Fresnel Prism and Lens Co., Bloomington, MN) embedded into the carrier lens were developed (Fig. 1A). Note that we measured (as described below) the press-on prism rated as 40Δ to provide 40Δ of deflection at the primary position of gaze, whereas the rigid PMMA Fresnel rated at 40Δ only provided about 36Δ. Throughout the rest of the article, we use these measured values when referring to these two types of prisms, respectively.

The original “horizontal” design of the peripheral prisms (so called because they provided only horizontal prismatic effect) was evaluated in four clinical studies (total 90 patients).^1^,^5^–^7^ At least two thirds of the patients perceived the peripheral prism glasses to be beneficial, usually reported as better ability to avoid obstacles on the hemianopic side. However, the horizontal peripheral prisms provide little help for driving, an important rehabilitation goal for many patients with hemianopia.^8^ Although the nominal field expansion of 20 degrees with the 36Δ prisms would cover most of the needed field for driving, there is a vertical gap of about 30 to 40 degrees between the upper and lower areas of peripheral field expansion (Fig. 1C). This variability is caused by individual differences in the distance between the spectacle plane and the patient’s eye. Thus, the expansion falls largely outside of the field of view seen through the windshield of a standard passenger car^2^ (∽30 degrees height) as shown in Fig. 1C.

The vertical separation of the expansion areas could be decreased by reducing the gap between the two prism segments. However, as the gap is reduced, central double vision may result if the prisms intersect the line of sight during head bobbing motions when walking or sitting in a moving car. This central visual confusion is annoying, and avoiding it was a major aim of the peripheral prism design.^1^ The minimum interprism separation that could be tolerated when walking was found to be 11 mm (median) in a multicenter study.^6^ To accommodate the majority of patients, the current fitting procedures use a standard interprism separation of 12 mm (about 33 degrees).^9^ With this separation, the view through the car’s windshield falls mostly within the gap between the two field expansion areas.

A new type of peripheral prism, the *oblique* design, invented by Peli,^10^ closes the gap between the field expansion areas while still maintaining the prism-free area in the center of the carrier lens. However, as detailed below, the oblique design reduces the magnitude of the lateral field expansion relative to that achieved with the same power of prism in the horizontal design. This limitation could be counteracted by use of higher-power prisms. We, therefore, describe and analyze here a number of novel optical designs for that purpose. In evaluating the various designs, we considered the impact of the prism power on the lateral field expansion as well as two other important properties: the apical prism scotoma and the eyes’ effective scanning range.

Apfelbaum et al.^3^ pointed out that the scotoma (blind area) that exists at the apex of a prism can have both positive and negative effects on the use of peripheral prisms. The apical scotoma reduces the annoying and useless diplopic effect from a unilaterally fitted prism. However, if the extent of the apical scotoma is wider than the angular distance from the primary direction of gaze to the apex, it results in a paracentral *binocular* scotoma.

Patients with hemianopia may compensate for their hemifield loss by visual scanning with head and eye movements toward the blind side. In this article, we only analyze the effect of eye scanning (i.e., the eye-in-head angle). The extent of the scanning range is an important consideration when evaluating different prism configurations. In the early implementation of the peripheral prism of moderate power (40Δ≈22 degrees), it was observed that the peripheral field expansion effect extended farther into the blind side when the patient’s eye scanned toward that side (up to 13 degrees toward the prism base), a very desirable property. Jung and Peli^11^ noted, however, that, with higher-power prisms, the extent of visual field with eye scanning toward the blind side was severely limited by total internal reflection (TIR). For example, the benefit from eye scanning to the blind side with 57Δ prisms is limited to about 5 degrees of scanning. The ideal high-power oblique prism design would have less restrictive extension with eye scanning, allowing the patient to combine the benefits of the prism deflection power and the eye scanning.

## OBLIQUE PERIPHERAL PRISM DESIGN

**FIGURE 2:**
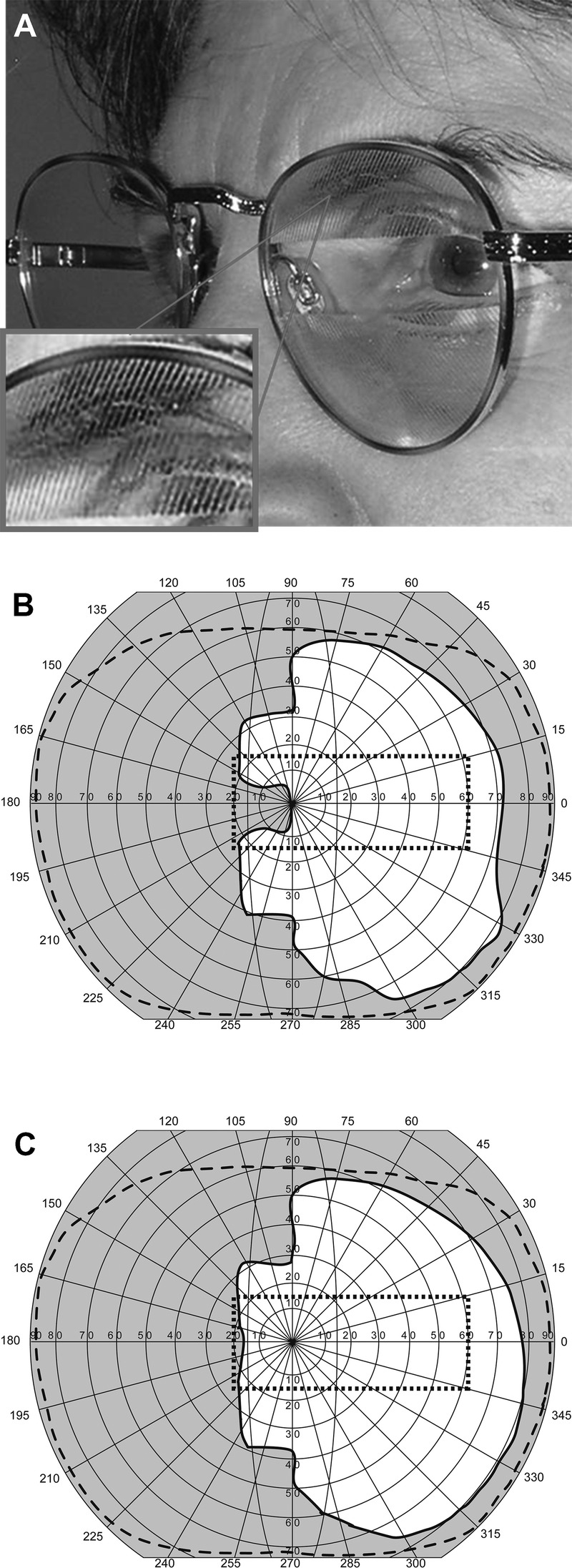
(A) Peripheral prism glasses in the oblique design constructed with press-on Fresnel prisms of 40Δ (see magnified inset) above and below the pupil mounted on the back of the left lens only for a patient with left hemianopia. The upper segment is oriented with the base out and down and the lower segment with the base out and up. (B) Binocular visual field of a patient with left hemianopia with oblique press-on Fresnel 40Δ prisms with the apex-base axes at 30 degrees tilt and with 11-mm interprism separation. The vertical gap between the expansion areas is reduced compared with the horizontal design (Fig. 1C), but the lateral field expansion effect is a little smaller. Note that the field in Fig. 1C was measured with 36Δ prisms. (C) Binocular visual field of the same patient with the same prisms but with a separation of 9 mm, which further reduces the gap. The dotted rectangle indicates the field of view through the windshield of a typical car.

In the oblique peripheral prism design,^10^ the apex-base axes of both the upper and lower prism segments are tilted such that the bases are closer to the horizontal meridian but without changing the positions of the prism segments or their shape (Fig. 2). The tilt creates a vertical prismatic effect that moves the field expansion areas vertically toward the horizontal midline of the visual field (reducing the central gap between the field expansion areas) while the prism segments remain in their peripheral location. Although the expansion is in more central areas of the field than with the horizontal design, the images shifted by the prisms continue to fall on approximately the same peripheral retinal locations, leaving the central retinal area unobstructed and unaffected by central double vision.

## IMPACT OF PRISM POWER AND PRISM TILT ON VERTICAL SHIFT AND HORIZONTAL EXPANSION

**FIGURE 3:**
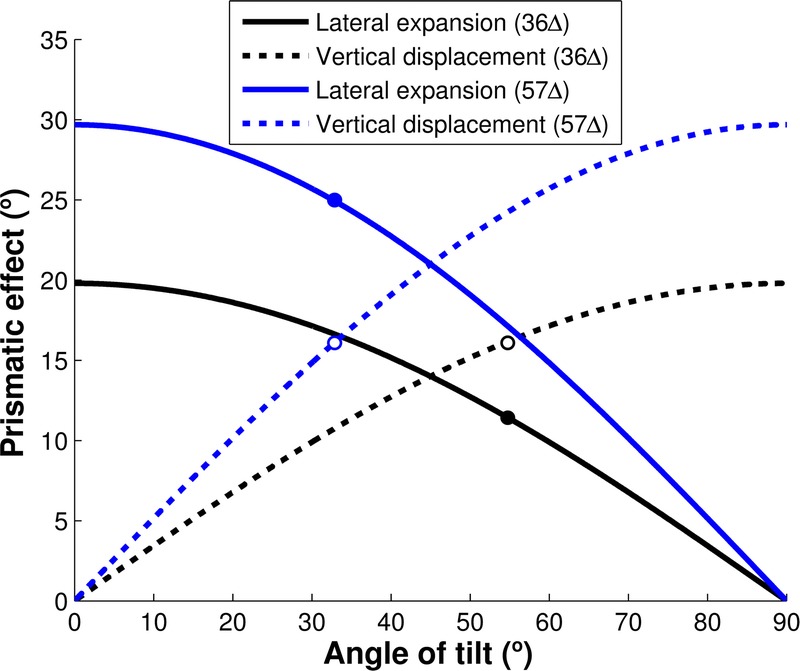
The trade-off relationship between oblique tilt angle and the lateral expansion and vertical displacement. Increasing the tilt angle of the base-apex axis increases the vertical shift (dashed curves) but reduces the lateral expansion (solid curves) for 36Δ (black curves) and 57Δ (blue curves) PMMA prisms. For small angles of tilt, the gain in vertical shift (gap reduction) is higher in magnitude than the loss in lateral expansion. To close the standard 12-mm interprism separation (equivalent to 33 degrees vertical gap between the prism expansion areas or 16.5 degrees for each prism segment), the 36Δ prisms require 56 degrees tilt angle (black open marker), which reduces the lateral expansion to only 11 degrees (black filled marker). However, the 57Δ oblique prisms can close the gap with just 34 degrees tilt (blue open marker) and still provide 25 degrees lateral expansion (blue filled marker).

Tilting the prism apex-base axis by angle β° shifts the expanded field segment vertically toward the center by a visual angle of p·sin(β°), where p is the rated prism power expressed in degrees. The same tilt, however, also results in a reduction of the lateral field expansion by a visual angle of p·cos(β°). The trade-off between the vertical shift and lateral field reduction is shown in Fig. 3 for 36Δ (∽20 degrees) and 57Δ (∽30 degrees) PMMA prisms. Because the vertical shift and the reduction of lateral field expansion are not linearly related, a smaller tilt angle can provide considerable vertical shift with only a small reduction of lateral field expansion. For example, a prism tilt of 30 degrees of the 36Δ prism provides 10 degrees vertical shift with only 3 degrees reduction (17 degrees) in lateral field expansion. However, 10 degrees vertical shift (total of 20 degrees for the top and bottom segments) from a 36Δ oblique prism of 30 degrees tilt is not sufficient to close the gap for prisms at the standard 12-mm (33 degrees) interprism separation.^9^

Further increasing of the tilt angle beyond 30 degrees could also reduce or eliminate the gap, but the magnitude of the lateral field expansion shrinks rapidly (Fig. 3). Although 56 degrees tilt of a 36Δ prism could close the gap with 16.5 degrees vertical shift (black open marker), it reduces the lateral expansion to only 11 degrees (black filled marker). The gap could be also reduced by fitting the prisms closer together on the carrier lens as illustrated in Fig. 2C for 40Δ press-on prisms. In this case, with 30 degrees tilt and 9 mm interprism separation (∽25 degrees separation at 20 mm from the nodal point), there should still be a gap of about 3 degrees as each segment produces a vertical shift of about 11 degrees (22 degrees total). Fig. 2C suggests total elimination of the gap; this small difference probably represents a limitation of the perimetric measurement accuracy. However, reducing the gap by bringing the two segments closer is not a practical solution. It may result in occasional central visual confusion as bobbing head movements swing the line of sight through the prism segments and only a small minority of participants (13 of 39; 33%) tolerated interprism separation of 9 mm or less.^6^ There are three different reference points that could be applied: the center of rotation of the eye, the nodal point, and the center of the entrance pupil. However, for simplicity, we use the nodal point as the reference for all calculations; any differences between using the nodal point and the other reference points are clinically negligible.^11^ The center of the entrance pupil is usually used as a reference point when calculating field of view in object space in camera systems, enabling perspective to be maintained for panoramic cameras and zoom lenses. However, because the rotation of the eye does not take place around the center of the entrance pupil (as in a panoramic camera), the nodal point that retains angular magnification is a better choice. Furthermore, we are interested in the perceived (retinal/image space) angular separation between two prism segments rather than the object-space field of view. To confirm that the nodal point is the relevant reference point to use, we perimetrically measured the perceived angular separation between two prism segments from a 1-m distance. With 12-mm interprism separation and 13-mm back vertex distance, the subject perceived 35 degrees angular separation. Thus, the reference point of the angular separation had to be located about 19 mm behind the prism (6 mm behind the cornea). Because the nodal point is 7.1 mm (6.5 mm when accommodated) and the entrance pupil is 3 mm (2.7 mm when accommodated) behind the cornea based on the Gullstrand model eye,^12^ the eye’s nodal point seems to be a better choice for the perceptual reference point of the angular separation than the center of the entrance pupil.

Increasing prism power, p, increases both the lateral field expansion and the vertical displacement of the expansion areas, meeting both needs. When the oblique design was first conceived^10^ and used,^13^ 40Δ was the highest power available. With 57Δ prisms, the highest power currently available, a 34-degree tilt angle is required to completely close the gap (blue open marker), which results in 25 degrees lateral field expansion (blue filled marker). We usually aim to leave about a 3-degree gap between the upper and lower field expansion areas to avoid any overlap of the expanded visual fields. Therefore, approximately 15 degrees vertical shift is required from each prism segment at a standard 12 mm (33 degrees) interprism separation.

## THE OBLIQUE PERIPHERAL PRISM IN CURRENT USE

Oblique peripheral prisms have been evaluated in a number of clinical studies providing evidence of their efficacy for walking^9^ and driving.^13^,^14^ In a multicenter randomized crossover trial with 73 patients, there was a clear preference for real (57Δ) over sham (<5Δ) prisms. In a study of on-road driving,^13^ the proportion of satisfactory responses to unexpected blind side hazards was better with the real than sham oblique prisms. Finally, in an ongoing driving simulator study, detection rates for pedestrian hazards on the blind side are higher with than without the oblique 57Δ rigid PMMA prism glasses.^14^

**FIGURE 4:**
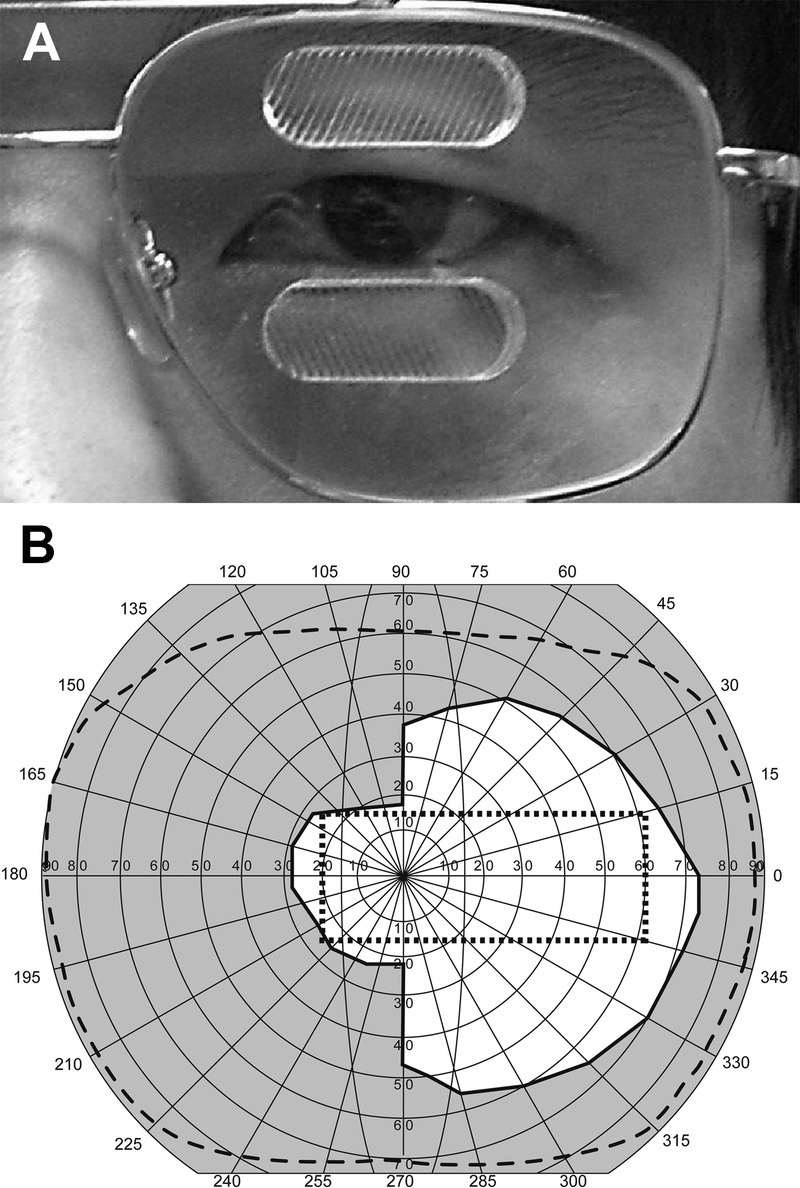
(A) Commercially available rigid PMMA oblique peripheral prism of 57Δ embedded in the left lens for a patient with left hemianopia. (B) Binocular visual field of a patient with left hemianopia wearing oblique prisms of 57Δ (30 degrees tilt angle) with a 12-mm interprism separation. Lateral 26 degrees field expansion and vertical 15 degrees shift were achieved with each segment; the gap between the prism expansion areas was eliminated. Note that the far peripheral field of this patient is smaller than a normal visual field (dashed line), yet the field expansion achieved by the oblique prism covers the view through the car windshield.

Although the high-power 57Δ Fresnel prisms (Fig. 4A) seem to address the needs of the oblique peripheral design in terms of closing the gap while still providing substantial lateral expansion (Fig. 4B), TIR from the high-power prisms may limit the benefit of scanning into the blind hemifield.^11^ Therefore, to maintain wide field expansion with a wide eye scanning range to the blind side, other solutions to achieve high-power oblique prisms need to be considered.

## METHODS TO INCREASE PRISM POWER

Because this article focuses on designs for high-power prisms, all Fresnel prism configurations use outward prism serrations, with the serrations facing away from the eye. Configurations with serrations toward the eye are not suitable (see Jung and Peli^11^ for detailed consideration).

### Yoked Prisms in the Carrier Lenses

**FIGURE 5:**
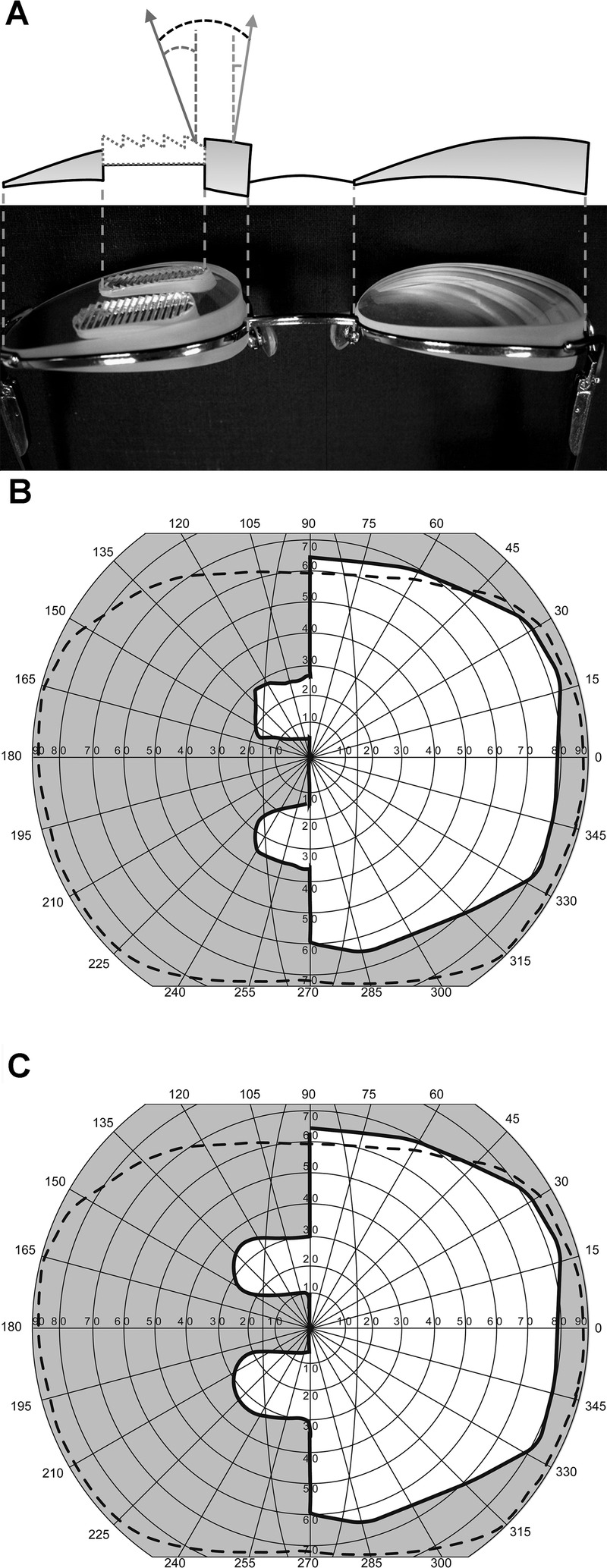
Yoked prism carrier lenses for increasing the effective lateral prismatic power and field expansion. (A) Horizontal peripheral prism glasses (rigid PMMA 36Δ) for left hemianopia embedded in one of the yoked ophthalmic prism carriers base right (10Δ). The prismatic effects of the carrier and peripheral prism sum up. (B) The binocular field of a patient with left hemianopia wearing 36Δ peripheral prism glasses showing about 20 degrees expansion. (C) The binocular field of the same patient when wearing the glasses shown in A with an increase in expansion of about 6 degrees (10Δ). The vertical difference in the positions of the expansion areas between B and C is an artifact of different head positioning in the perimeter. The same effect of head tilt is in play in daily use of the peripheral prism glasses.

Yoked prisms are full aperture prisms placed in front of each eye that have the same power and base direction (e.g., bases both to the right or the left). Yoked prisms with base in the direction of the field loss have been proposed as an optical treatment for hemianopia,^4^ although they do not expand the field or even shift it, as eye movements counter their effect.^3^ However, when yoked prisms are used as carriers for the rigid peripheral prisms, which are embedded in a pocket in the carrier lens, and their base is in the opposite direction to that of the peripheral prisms as shown in Fig. 5A, then the total prismatic effect of the peripheral prisms and the carrier lenses is greater than the peripheral prisms alone. As illustrated in Fig. 5, where yoked carrier prisms [10Δ (∽6 degrees), base right] were combined with the 36Δ (∽20 degrees) rigid PMMA Fresnel horizontal peripheral prisms, base left, the prismatic effects sum up algebraically, resulting in a total lateral field expansion of 46Δ (∽26 degrees) (Fig. 5C). The additional 6 degrees of lateral expansion compensates for loss of lateral extension caused by the increased tilt angle needed to close the gap between the prism segments. A 36Δ Fresnel prism with 39 degrees tilt fitted into a 10Δ yoked prism provides 20 degrees lateral expansion with 16.5 degrees vertical displacement. This is sufficient to close the gap even with the standard 12-mm interprism separation while keeping the lateral expansion and eye scanning range similar or better than that possible with a 36Δ peripheral prism in the horizontal design. However, the scanning range is still limited with the higher-power prism even if the additional lateral field expansion is achieved by the yoked prism in the carrier lenses. Note that this approach requires drilling of the yoked carrier lens and embedding a rigid PMMA prism (Fig. 5A). By comparison, applying press-on prisms to a yoked carrier lens will not result in any increase in the prismatic effect of the peripheral prisms.

### Bi-Part Double Fresnel Prism

**FIGURE 6:**
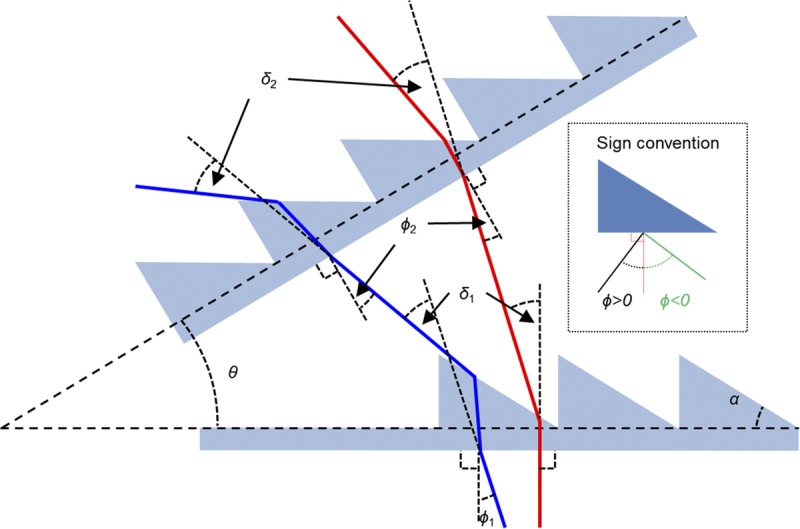
The rays’ paths through a bi-part double Fresnel prism. The two Fresnel prism segments with apical angle *α* are inclined relative to each other at angle *&thetas;*. The total deflection of light *δ* is the sum of the deflection powers of the two segments (*δ* = *δ*_1_ + *δ*_2_). As with a conventional prism, the effective prism power of the bi-part prism is increased by a negative angle of incidence and is limited by TIR (blue ray).^11^ This design offers some flexibility through a trade-off between a wider eye scanning range and a higher nominal power (see Fig. 9). When the angle of incidence increases, the effective prism power of the bi-part prism increases (from red rays to blue rays on the left). If the eye scanning angle in the first prism (closer to the eye) is just under the critical angle of incidence (blue rays), the angle of incidence in the second prism should be higher than the critical angle of incidence to prevent TIR (blue rays).^11^ This is achieved by increasing angle *&thetas;*.

Another way to increase the power of the peripheral prism is to combine (stack) two Fresnel segments one over the other. If the two prisms are stacked parallel to each other, the angle of incidence into the second one is negative and high enough to lead to TIR and loss of transmission.^11^ Therefore, the two segments have to be angled relative to each other to reduce the angle of incidence and enable transmission as shown in Fig. 6.

For convenience, we ray trace through the bi-part prism as if the rays were emerging from the eye rather than from the object of regard. This is particularly useful as one can start from the foveal line of sight that represents the most extreme ray that will fall on the functioning side of the retina of a person with hemianopia after deflection by the prism. The deflection angle *δ*_1_ of the prism next to the eye, with refractive index *n*, angle of incidence *ϕ*_1_, and apical angle *α*, is as follows:


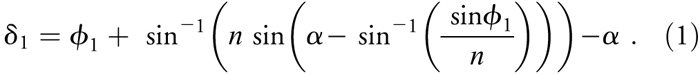


Because of the angle, *&thetas;*, between the prisms, the angle of incidence at the second prism *ϕ*_2_ and its deflection angle *δ*_2_ are derived as follows:


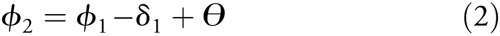



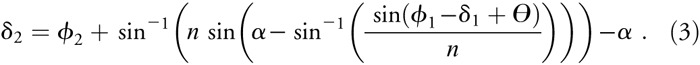


The total deflection angle of the bi-part prism is *δ* = *δ*_1_ + *δ*_2_.

**FIGURE 7:**
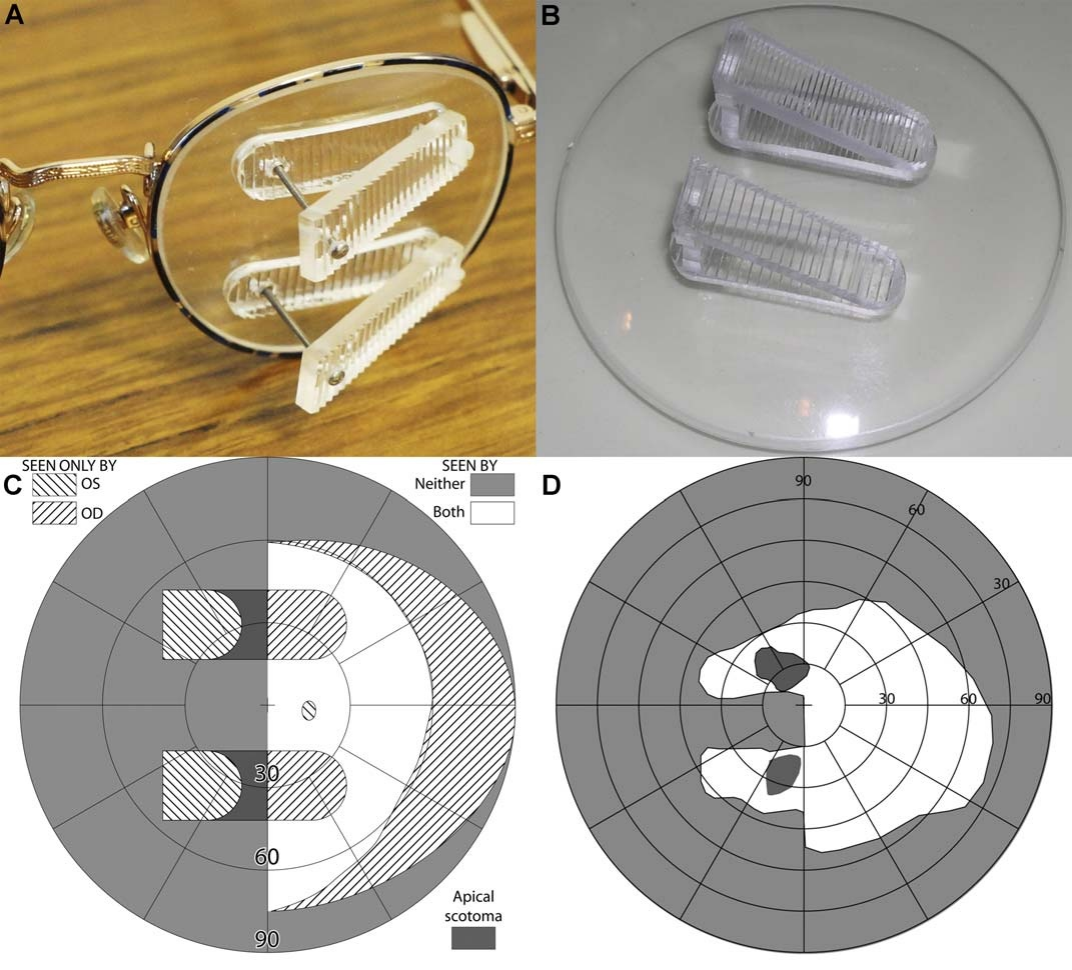
Bi-part prisms segments. (A) Spectacle-mounted bi-part system with two prisms of 36Δ and an adjustable screw mount enabling adjustment of the angle between the two prisms. (B) Bi-part prism constructed with a fixed angle between the two prisms, which could be used in prescription production. Note here that one segment (lower) is constructed from 36Δ Fresnel prisms and the other with 57Δ prisms. (C) Calculated binocular Goldmann visual field^3^ for a patient with left hemianopia wearing the bi-part horizontal peripheral prisms shown in A with 29 degrees between the two prisms. The large apical scotomas extend into the left hemifield, creating paracentral scotomas in the binocular field. (D) Measured binocular visual field of a patient with left hemianopia wearing bi-part prism glasses. The paracentral scotomas are apparent in the binocular field. This patient has some overall reduction of sensitivity peripherally in addition to the hemianopia.

We constructed a bi-part prism using two PMMA 36Δ rigid Fresnel prisms with a screw adjustment to vary the angle between the prism segments (Fig. 7A) and measured the amount of deflection for four angles covering a practical range. The rated prism power is the calculated deflection angle of a prism for a given refractive index and apical angle, assuming normal incidence at the first (near eye) surface. The measured prism power is quite sensitive to variations in the angle of incidence.^11^ To measure the prism power at normal incidence, a laser pointer was aligned to be perpendicular to the flat surface of the Fresnel prism mounted on a rotational stage. First, the stage was adjusted so that the beam was reflected back to the pointer from that surface without any lateral deviation. The position of the deflected ray was then marked and compared with the position of the undeviated ray when the prism was removed.

**FIGURE 8:**
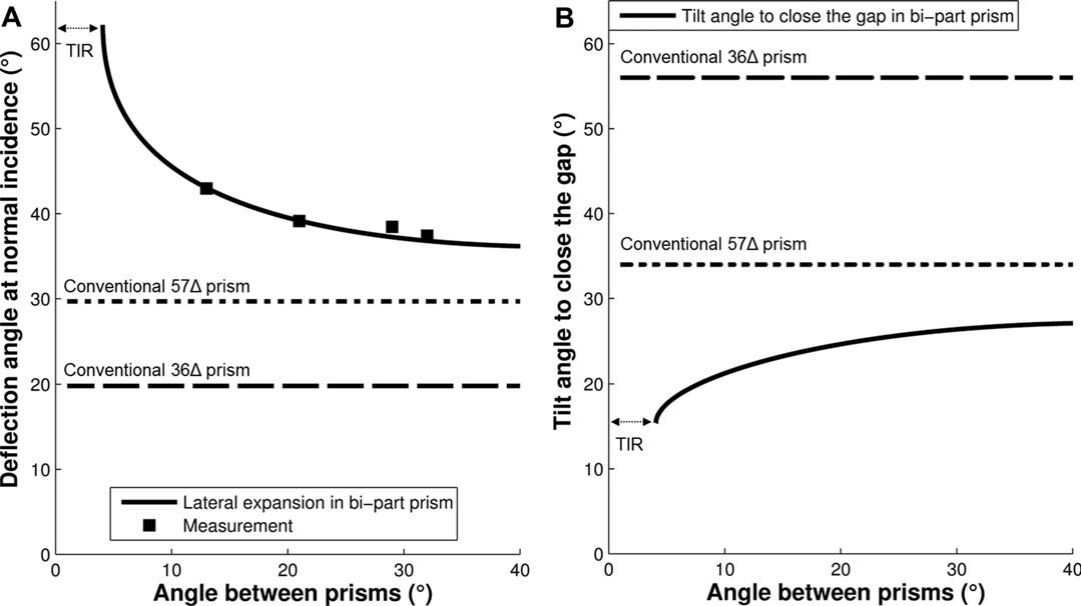
(A) The calculated (solid line) and measured (solid squares) deflection angles, at normal incidence, of a bi-part prism constructed from two PMMA Fresnel prisms of 36Δ as a function of angle, *&thetas;*, between them. The nominal deflection angle (normal incidence) for a bi-part prism converges to a fixed value of about 38 degrees (∼78Δ) for angles between the prisms larger than 29 degrees. As the angle between the prisms is reduced, the deflection angle increases rapidly and the transmittance also decreases rapidly toward the critical angle (angle of TIR). Rated prism powers of conventional 57Δ (dotted line) and 36Δ (dashed line) are illustrated. (B) The tilt angle for the oblique design (with 12-mm interprism separation) needed to close the gap (33 degrees) between the expansion areas as a function of the angle between the bi-part prisms. The tilt angle required to close the gap with conventional 57Δ (dotted line) and 36Δ (dashed line) are illustrated for comparison.

Fig. 8A shows the calculated prism powers at normal incidence (deflection angles) for bi-part prisms composed of two 36Δ Fresnel prisms as a function of the angle between the prisms as well as four measurement results. The measured deflection matches the calculated deflection well. With the 36Δ prism segments and an angular separation of 29 degrees, we achieved a high deflection of about 38 degrees (∽78Δ). With a 13-degree angular separation, the deflection power increased to 43 degrees (∽93Δ). Note, however, that there is a trade-off between the effective prism power and the transmittance. As the angle between the prisms is reduced, the prism power at normal incidence increases rapidly, but the light transmittance also decreases rapidly. Fig. 8B shows the required tilt angle to close the gap between the expansion areas for oblique design bi-part prism segments with a standard 12-mm interprism separation compared with conventional 57Δ and 36Δ prisms.

Having calculated and measured that we could achieve a deflection of 38 degrees with a bi-part prism, we then calculated^3^ (Fig. 7C) and also measured (Fig. 7D) the resulting visual field expansion for a patient with left hemianopia fitted with a unilateral bi-part peripheral prism in the horizontal configuration (as shown in Fig. 7A). Using Goldmann perimetry (V4e target), the measured lateral visual field expansion was, as expected, about 40 degrees into the blind left hemifield under binocular viewing conditions. However, two paracentral scotomas were also apparent within the areas of visual field expansion (Fig. 7D). These were caused by the large apical scotoma associated with the high power of the bi-part prism extending into the visual field expansion areas where it could not be compensated for by the seeing visual field of the nonprism eye. The extent of the measured apical scotoma is larger than the calculated one in part because the lens was constructed with the apex mounted into the carrier too far temporally (Fig. 7A) than it could and should have been.^11^

**FIGURE 9:**
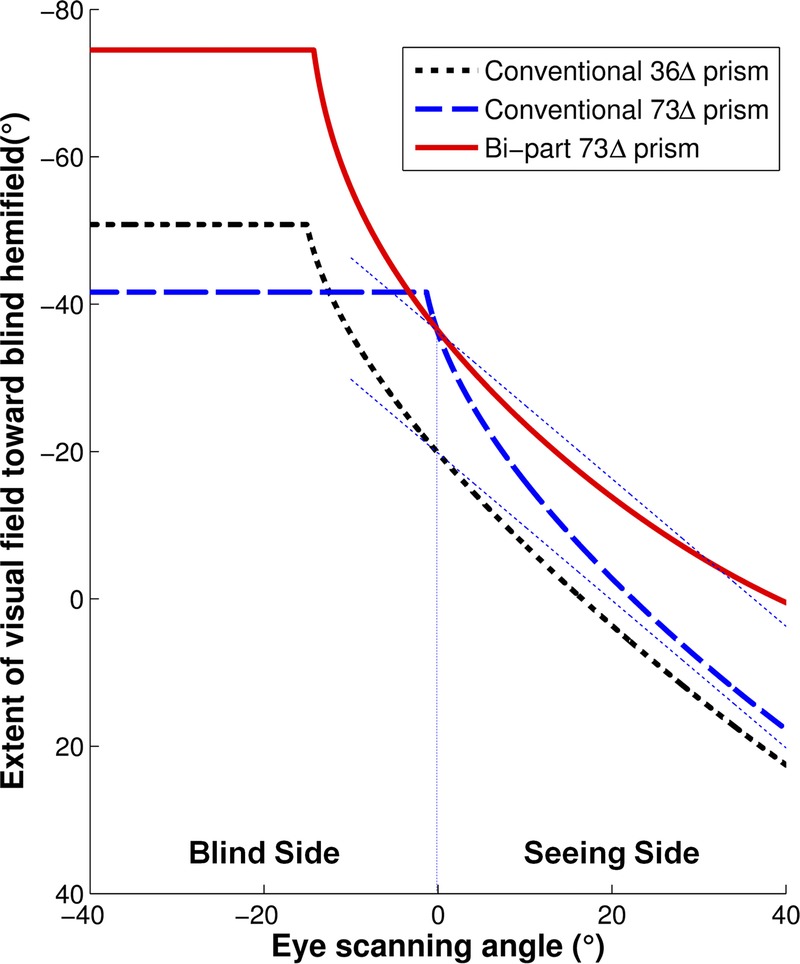
A comparison between bi-part and conventional prisms of the calculated extent of visual field as a function of eye scanning angle. For a patient with hemianopia, the extent of visual field is from primary gaze (eye scanning angle = 0 degrees; head direction) toward the blind side or prism base. At the foveal line of sight, the angle of incidence is equal to the eye scanning angle. The effective eye scanning range is limited by the angle of incidence, resulting in 50% transmittance. Within this range, the effective prism power increases as the patient scans farther toward the blind side. As a result, the increase in the extent of the visual field is larger than the scanning angle, representing prism minification (image compression). The increase in the extent of the visual field saturates when the eye scanning angle exceeds the critical angle. On eye scanning into the seeing side, the extent of field toward the blind hemifield is largely unchanged with the bi-part prism even when compared with the conventional 36Δ prism, and it is much more stable than with the conventional high-power prism. Compare with the thin dashed lines that represent a constant field extent at all scanning angles.

In addition to the high prism power, the bi-part prism enables a beneficial wider eye scanning range than a conventional prism with the same high power. Fig. 9 shows a comparison of the extent of visual field relative to the straight ahead head position as a function of the eye scanning angle for bi-part versus conventional prisms; the eye scanning angle is the same as the angle of incidence. For much of the eye scanning range, the extent of visual field toward the blind hemifield is determined by the summation of the eye scanning angle and the deflection angle at the foveal line of sight (which is a nonlinear function of the eye scanning angle). As the patient looks toward the blind hemifield (negative eye scanning angles), the extent of field to the blind side increases rapidly because of the increase in the effective prism power with the increasingly negative angles of incidence. When the eye scanning angle exceeds the critical angle of incidence and TIR occurs (e.g., at about −14 degrees for the bi-part prism), the extent of visual field saturates at the value obtained at the critical angle. Any further increase in eye scanning angle just changes the retinal eccentricity but does not affect the extent of visual field toward the blind hemifield.

The critical angle of incidence, where TIR occurs, provides the maximal theoretical effective prism power with the widest field expansion. However, the transmittance at the critical angle of incidence is zero. In consideration of the trade-off between the effective prism power and transmittance (see Fig. 3 in Jung et al.^11^), we consider the maximal practical effective prism power as the effective prism power at the angle of incidence that results in 50% transmittance. This is consistent with the commonly applied half-maximum luminance rule in calculating the field of view of a low-vision telescope.^15^ With 36Δ (∽20 degrees) conventional prism, the theoretical maximum prism power is 92Δ (43 degrees), reached at the critical angle of incidence (−15.7 degrees) with no light transmission. The prism power associated with 50% transmittance, reached at −15 degrees angle of incidence, is 73Δ (36 degrees). The bi-part prism may be thought to have a lower transmittance than the single conventional prism and, therefore, a narrower scanning range (up to the angle of incidence that causes 50% transmittance) because of transmission losses through two prisms. However, because the transmittance variation with the angle of incidence is so steep around the critical angle of incidence, the effective eye scanning range of the bi-part prism calculated using the angle of incidence that results in 50% transmittance is almost the same as the scanning range of the single prism. For the case shown here (Fig. 9), the effective scanning range is reduced only from 15 to 14.3 degrees.

As can be seen in Fig. 9, the effective scanning range (up to the point of TIR) with the 73Δ bi-part prism is close to its lower power 36Δ component and wider than the scanning range for a conventional 73Δ prism. The extent of visual field toward the blind hemifield possible with the bi-part prism is also wider than with the conventional 73Δ prism by about 14 degrees. Thus, a bi-part prism enables a wider range of scanning and a larger extent of visual field to the blind side at most positions of eye scanning angle than a single prism of the same power. The wide expansion with both conventional and bi-part prisms results from the minification caused by the rapid change of effective prism power on approach to the critical angle of incidence.^11^ That high minification (compression) may limit the visibility of small objects at the far end of the expanded field. Other configurations of the bi-part prism are possible by turning one or both of the Fresnel components so that the serrations are toward rather than away from the eye. However, none of them is practical because of either low power or intense spurious reflections.^11^

### Mirror-Based Periscopic Prism

**FIGURE 10:**
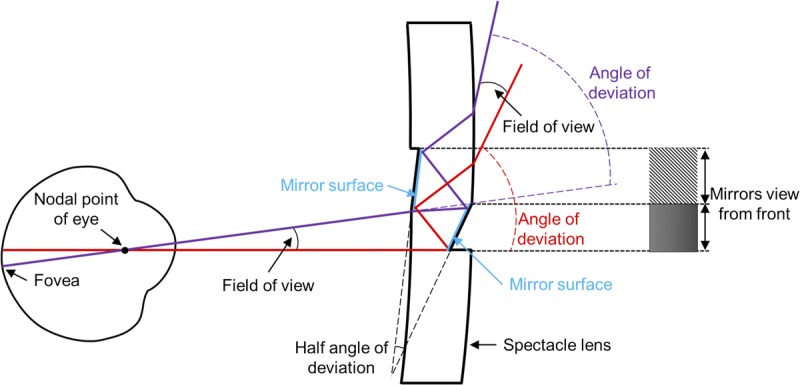
Spitzberg’s mirror-based periscopic design viewed from above. Together, the two mirrors form a ray-shifting device that is similar to a prism in terms of the image shift. In this illustration, the thickness of the spectacle lens is 5 mm and the angular difference of the slanted mirror surfaces is 17 degrees, giving a prism power of about 67Δ, but the field of view is limited to only about 5 degrees.

**FIGURE 11:**
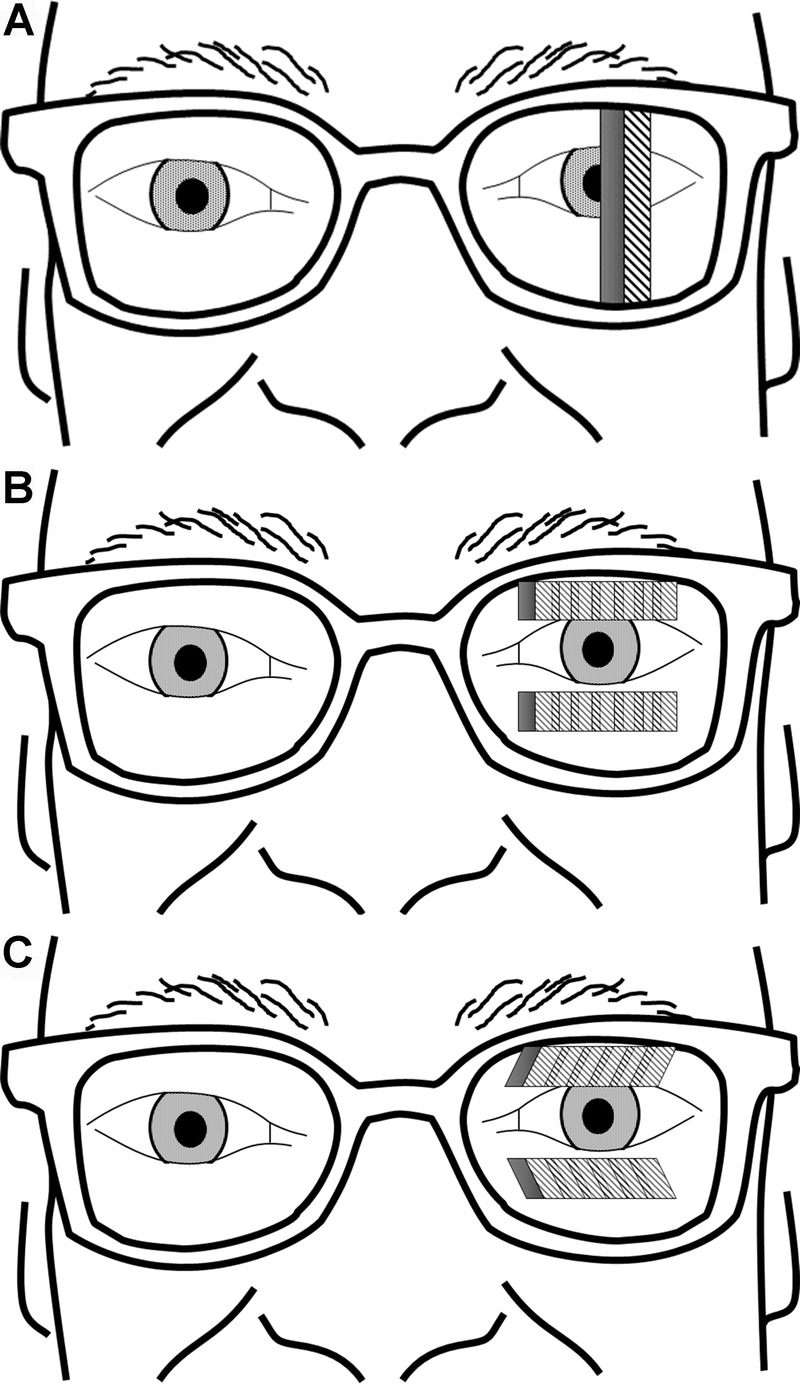
Various configurations of the mirror prism-like elements used as a field expansion device for homonymous hemianopia. (A) Spitzberg’s mirror prismatic device was designed as a single element unilateral sector prism field expander that extends vertically across the whole lens. While the prismatic deflection angle can be large, the field of view seen through this “prism” is narrow and constrained by the thickness of the carrier spectacle lens. (B) The field of view can be expanded by creating a Fresnel-like structure with segments of these elements placed one next to the other. Short elements of this type can then be used to implement the peripheral prism design. (C) Tilting the same series of mirror elements can be used to implement the oblique peripheral prism design with a higher deflection power.

A third approach to increasing the prism power is a Fresnel prism–like device constructed of pairs of mirrors, inspired by a design that Dr. L. Spitzberg proposed (personal communication) for a prism-like double mirror-image shifting device to be used as a classical sector prism for hemianopia, as illustrated in Figs. 10 and 11A. Sector prisms for hemianopia are typically restricted to 20Δ in power because they affect foveal vision. The reduced optical quality and increased lens thickness of higher-power ophthalmic prisms limit their use in obtaining a greater deflection (shift) of the image through the prism.^3^ Spitzberg proposed using a pair of mirrors embedded in the spectacle lens in a periscope-like arrangement but not parallel to each other as in a typical periscope. The angular shift of the two mirrors results in an image deflection angle that is about double the angle between them. We call this design reflective prism. In addition to the large angle of deviation that could be achieved with such a system, it is mostly free of chromatic dispersion because of the use of mirrors, which is considered the main cause of reduced image quality in ophthalmic prisms.^16^,^17^ There is also no image reversal caused by the two reversing reflections through the system.

Although Spitzberg’s design was inspiring, it had a number of limitations. The original design considered only the reflections at the mirror surfaces and ignored the refractions at the two other surfaces as the light enters and exits the carrier lens (shown in Fig. 10), which would reduce the total angle of deflection by a small amount from the angle of deviation caused by the mirrors alone. A mirrors-only design would be possible only if the mirrors were in air and suspended somehow at their edges. More importantly, the design did not consider the physical limitations of the spectacle lens thickness on the field of view available through such a device. With a carrier lens as thick as 5 mm, the shifted field of view through the device would be too small (∽5 degrees) to be of any practical use (Fig. 10). Peli^10^ proposed to expand the field of view by combining a series of these limited field-of-view reflective prism elements into a Fresnel prism–like device, as shown in Fig. 11, that could be implemented as peripheral prisms in either the horizontal or oblique design (shown schematically in Fig. 11, B and C).

**FIGURE 12:**
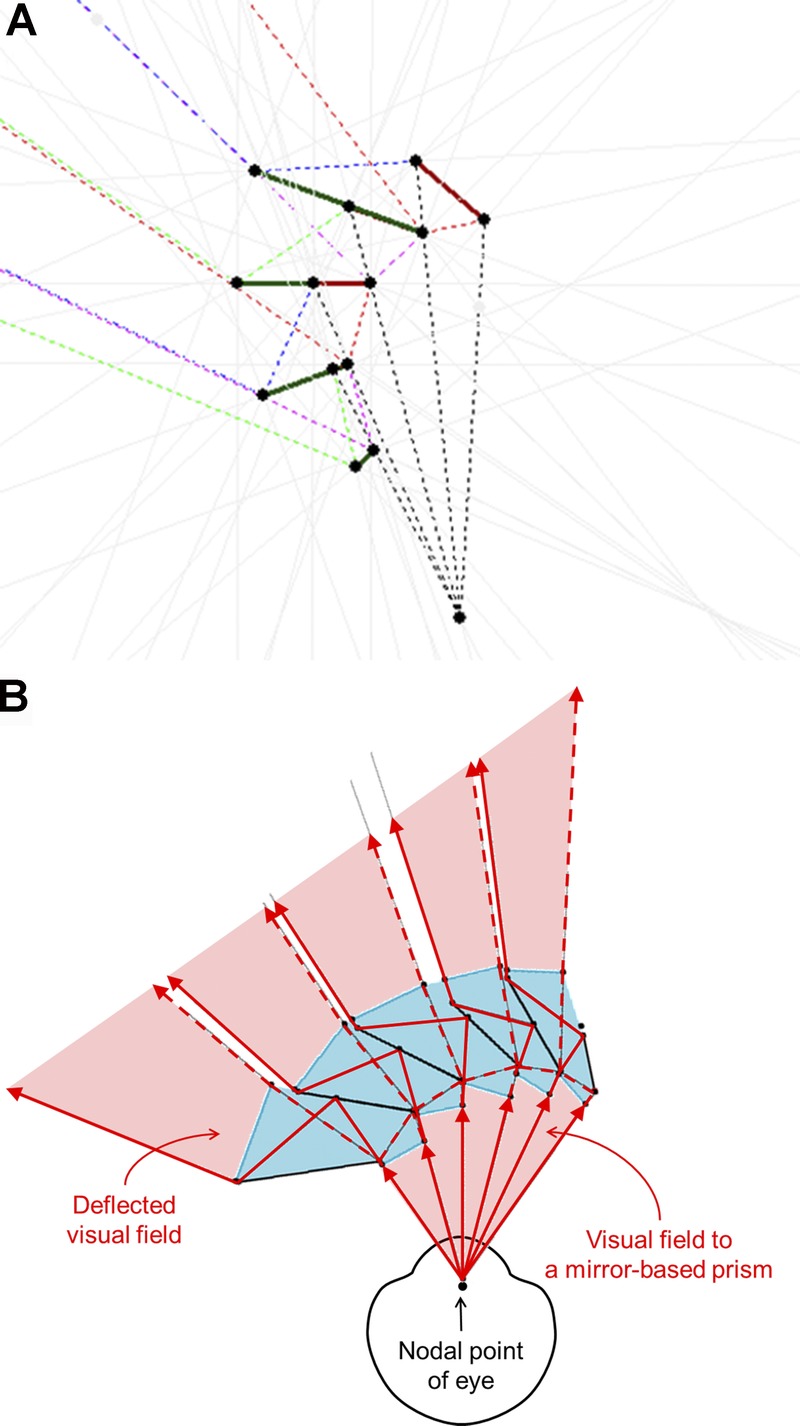
Ray tracing of the field of view constraints of a reflective Fresnel prism design. (A) The mirrors-in-air design is restricted in extent by the limitations imposed by the field of view of each segment (because of lens thickness) and the increased angular rotation of the second prism in every pair, which leads to diminishing size. Shown here for approximately 40 degrees of deflection. The solid lines are mirror segments, and dashed lines are traced rays. (B) A solid design in PMMA provides additional flexibility of design, but with additional complexity. It can result in a larger extent of the Fresnel prism section than the in-air design (A). In the implementation shown here, there are very small blind areas (shown here as white gaps) that, because of their parallel tunnel-field nature,^18^ result in rapidly diminishing and negligible effects at the distances of objects of interest.

**FIGURE 13:**
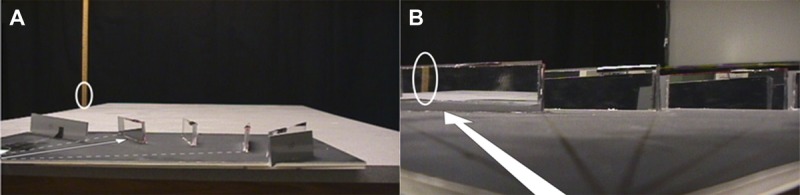
A reflective prism device demonstration unit built with double-sided mirror segments mounted on a dark surface. (A) A photo from the side showing the real target (wooden ruler) marked by a white ellipse. The user’s direction of view is shown by the thin white arrow drawn on the dark surface from left to right. (B) An image of the target is seen in the reflective prismatic device in the direction of the thick white arrow; it is shifted by 36 degrees (∼73Δ).

We have considered two implementations of the reflective Fresnel prisms: one (following Spitzberg’s original concept) constructed from mirrors in air connected and held in place by a structure above and below and the other a solid device constructed from PMMA with internal mirrored surfaces. The optics of the former is easier to describe and follow, but it might be harder to construct, particularly for the oblique design, and may be impractical to maintain and clean as a spectacle lens component. As shown in Fig. 12A, if such an element is constructed from mirrors silvered on both sides (in air), it can be designed to provide a uniform deflection power with no or minimal field interruptions. However, the design curves and converges quickly, enabling only a few Fresnel segments to be constructed, and thus it is also limited in the field of view that it can provide for the shifted images. This design was computed by using KSEG Free Interactive Geometry Software (http://www.mit.edu/∽ibaran/kseg.html). A large-scale prototype implementation of such a design is shown in Fig. 13, illustrating the layout and demonstrating the shifted view through such a device, achieving a large shift of 36 degrees, approximately 73Δ. The addition of PMMA material filling between the mirrors (Fig. 12B) increases the complexity of the design because of the effect of refraction and the possibility of TIR but offers increased flexibility of design. Additional PMMA surfaces can be used to expand the field of view through the device by moderating the convergence of the elements as shown in Fig. 12B. Such a device would be much easier to construct and maintain, can be easily cleaned, and is far less fragile. As a solid piece of plastic, it can be easily cut at different angles to serve in the oblique design.

## DISCUSSION

The horizontal and oblique peripheral prism configurations improve on prior prism designs for expanding the fields of patients with hemianopia.^3^ The oblique design has the advantage of closing the vertical gap between the upper and lower field expansion areas that remains with the horizontal peripheral prism design. This effect is particularly important for use in driving (Fig. 4B). However, the expansion of the field in the central area comes at a cost of a reduction in the lateral field expansion. Thus, higher-power prisms are desirable for the oblique design. However, higher-power prisms have some optical side effects such as TIR and a wider apical scotoma. We considered these in analyzing the higher-power devices proposed here.

In earlier articles, we claimed that the peripheral prisms, because of their location across the lens midline, provide the same expansion at any lateral eye scanning angle.^1^,^3^ This desirable property was true for the 40Δ that we used first and especially so for the press-on prisms that were mounted on the eye side of the spectacle lens.^11^ Later, we realized and verified that, with higher-power prisms, the additional expansion achieved with eye scanning toward the blind side (prism base side) may be severely limited by TIR.^11^ Some of the designs we presented here address this limitation and enable further eye scanning with expansion, even with higher powers. At the other end of the prism, there is the prism apical scotoma that is generally seen as a limitation of prisms in field expansion applications. However, in the case of unilateral peripheral prisms, it has the advantage that it can reduce or even eliminate peripheral diplopia. Although binocular confusion is essential for field expansion, diplopia provides no benefit.^3^ When the apical scotoma extent matches the angular span of the prism between primary gaze and the prism apex, the diplopia is completely eliminated.^3^ The apical scotoma extent is equal to the power of the prism at the apex (expressed in degrees). For a standard ophthalmic prism (or Fresnel prism), this power is affected by the angle of incidence between the eye’s nodal point and the prism apex.^11^ With high-power prisms, the apical scotoma can be so wide that it may extend toward the base past the optical center of the spectacle lens and therefore into the patient’s blind hemifield, resulting in a binocular scotoma (as in Figs. 7C & 7D), even with unilateral fitting and under binocular viewing.

The apical scotoma problem may be addressed by extending the prism size on the apex side and thus pushing the apex of the prism farther away from the blind hemianopic side. With the physical limitation of the interpupillary distance and the typical frame design that flares away from the nose at cheek level or lower lens edge, the maximal extent of the peripheral prism is limited to about 30 degrees for the lower prism segment. Therefore, a peripheral prism design that extends at least the upper prism segment farther nasally on the carrier lens on the side of the field loss may offer more flexibility. Another approach that may resolve this issue is to fit the prisms on the other lens (i.e., base-in peripheral prism on the right carrier lens for a patient with left hemianopia). In that case, the prism apexes are on the temporal side of the lens and can be easily extended much farther in that direction. Such placement and extension will work with any of the designs described here, but it will only be needed for prism powers where the horizontal components are substantially higher than the currently available 57Δ. For example, a 66Δ effective oblique prism with 30 degrees tilt angle can provide 55Δ of lateral expansion and 30Δ of vertical displacement and expand the field laterally without scotoma or vertical gap. However, to achieve 66Δ effective prism power at the apex, the nominal prism power has to be much higher than 66Δ, which will result in no eye scanning range into the blind side.

Adding 10Δ yoked prisms to carrier lenses is easy to produce and adequately compensates for the lateral expansion, which is lost as a result of the prism tilt. It is consistent with current production techniques. In addition, the apical scotoma is not enlarged by the carrier power and does not cause the mid-field binocular scotoma. However, 10Δ yoked prisms make the carrier lenses very thick (where, on the nasal side, it can be physically uncomfortable too), heavy, and with questionable cosmetic appeal. Although higher carrier lens prism powers are possible, they are not a practical solution. High-power full-field prism lenses also result in notable distortion on the base side of the prism.^19^,^20^ That distortion falls in the blind hemifield when yoked prisms are used with the base to the blind side to treat hemianopia^4^ (i.e., the distortion is in an area of the visual field where the prisms do not work^3^). However, when used as described here, the base, and thus the distortion, is in the residual field and therefore may cause discomfort to the user, especially for people who are prone to motion sickness. The optical quality centrally through the prism carrier is also somewhat reduced by the prism color dispersion.^16^,^17^

The bi-part prism can also be constructed with current technology. It can provide a much higher deflection power than currently available devices. More computational and experimental work is needed to determine optimal prism segment shapes and sizes and the angle between the two segments for an oblique design. The bi-part prism offers an interesting advantage as it relates to the range of eye scanning. As we have shown, a bi-part prism constructed from two 36Δ prisms only limits the benefit from scanning to 14 degrees as slightly less than a single 36Δ prism (15 degrees scanning range), despite the increase in power. The same increase in power for a conventional prism will severely restrict the additional field expansion with eye scanning to the blind side (2 degrees) by reducing the angle of incidence that leads to TIR. The bi-part prism may be adjusted to trade deflection power for scanning range to fit a specific design or application requirements. Of course, if a very high power is generated, the apical scotoma is similarly increased and the prism may have to be mounted on the lens contralateral to the field loss and extend sufficiently far temporally to limit the apical scotoma. In addition, the higher minification around maximum field expansion in a bi-part prism may cause a visibility issue.

The optical quality of the bi-part prism is relatively poor because it involves a double pass through Fresnel segments. Importantly, this design protrudes out of the carrier lens to such an extent that it is cosmetically poor, difficult to clean and maintain, and may be fragile. If it breaks when worn, it may even be dangerous, like a number of previously proposed mirror devices.^21^,^22^ A larger bi-part prism, as needed to limit the effect of the apical scotoma, will also protrude farther from the lens and will be more prone to breakage.

The reflective periscopic prism elements are simple to manufacture. Single segments of the system can be produced as a piece of PMMA machined to the required shape, two of the surfaces mirrored, and the assembly of all segments glued together to create the structure shown in Fig. 12B. This Fresnel prism–like structure can be cut to the required prism either horizontal or oblique at any tilt. The deflection power possible with this system is substantially higher than currently available prisms, making this design suitable for the oblique design with minimal tilt and with the additional advantages of a higher-power prism. Because most of the light shift is achieved with mirror deflections rather than refraction, the color dispersion is limited and therefore the optical quality is higher than that of refractive prisms. Whereas the system shown in Fig. 12B is not completely free of the limitation of TIR, the impact of the TIR is highly reduced. Therefore, the ability to scan with the eyes to the blind side while continuing to gain field expansion farther into the periphery is only slightly restricted with this design. However, this functionality requires that the peripheral prism extends to the blind side beyond the optical center of the carrier lens. This requires a wider Fresnel structure. As can be seen in Fig. 12, the basic design of this system curls and as a result may be limited in the number of segments that can be put together practically. This effect can be controlled to some extent by separating the elemental segments and rotating them slightly relative to each other. This will result in nonimaging sections (small gaps) between the periscopic mirror prisms. Yet such a high-power and high–image quality design may be effective despite these field interruptions. The limited size of the periscopic Fresnel system also reduces the ability to extend the prism into the apex side to reduce the impact of the apical scotoma. Here too, further design and experimental work is needed to determine the best option for specific applications.

The oblique peripheral prism is emerging as the solution of choice for field expansion in hemianopia, especially in considering the use in driving. For this application, a higher-power prismatic element is desirable. We have presented a number of options, each with its own limitations but also with numerous advantages in relation to secondary aspects of the main field expansion utility. The ideal design would provide wide lateral field expansion in areas of the field surrounding the horizontal meridian, would permit field expansion to follow when the user scans into the blind side, and would avoid, as much as possible, creating paracentral binocular scotomas secondary to the apical scotoma. These considerations apply to the current preferred mode of unilateral fitting with binocular viewing, which uses binocular multiplexing. Other modes for peripheral prism field expansion are possible,^3^ but with any mode, the full set of considerations needs to be reevaluated. The high-power prism devices we introduced here in consideration of treating homonymous hemianopia may find use in other field expansion applications.^23^,^24^

**Eli Peli**
Schepens Eye Research Institute
20 Staniford St
Boston, MA 02114
e-mail: eli_peli@meei.harvard.edu
